# A radiologic morphometric study of sellar, infrassellar and parasellar regions by magnetic resonance in adults

**DOI:** 10.1186/2193-1801-3-291

**Published:** 2014-06-09

**Authors:** Seizo Yamashita, Luis Antonio Resende, André Petean Trindade, Marco Antonio Zanini

**Affiliations:** Department of Radiology, School of Medicine, University of São Paulo State/UNESP, Kragujevac, SP Brazil; Department of Neurology and Neurosurgery, School of Medicine, University of São Paulo State/UNESP, Kragujevac, SP Brazil

**Keywords:** Anatomy, Sella turcica, Sphenoid sinus, MRI

## Abstract

**Objective:**

To evaluate variations of some anatomic structures of sellar and parasellar regions and their possible differences between genders and age groups.

Patients and methods: Magnetic resonance images (MRI) of 380 patients were performed to analyze the dimensions of the sphenoid sinus, pituitary gland, optic chiasm, intra-cavernous carotid distances, distance between columella nasal - sphenoid sinus; and columella nasal-pituitary gland. The patients age ranged between 20 and 80 years (mean age 48 years). The study included 235 females (mean age 53 years) and 145 males (mean age 40 years).

**Results:**

The transverse length of the pituitary, the inter-carotid distance and the height of the pituitary were similar between genders and age groups. The width and height of the optic chiasm showed differences only between females of different ages. Males presented greater distances between nasal columella and sphenoid sinus. The most common type of pneumatization of the sphenoid sinus was the sellar, and depending on the age group, sphenoid sinus was larger in males than females.

**Conclusion:**

The anatomy of the Sellar and parasellar regions is complex and varies widely within the normal range. They are a small area, rich in anatomical details affecting multiple physiological systems in the body and, therefore, have great importance in several medical fields. A better understanding of these complex structures is essential in clinical diagnosis and treatment of disease.

## Introduction

The sellar and parasellar regions are anatomically complex area composed by the sella turcica, pituitary gland and adjacent structures, which make their surgical access difficult.

The sellar area is bounded by sphenoid sinus anteroinferorly, the paired cavernous sinuses laterally, the suprasellar cistern and its contents, diaphragma sellae and hypothalamus superiorly, and the dorsum sella and brainstem posteriorly.

The pituitary gland is composed of two anatomically and functionally distinct lobes: the anterior and the posterior lobe.

The sella is part of the superior portion of the sphenoid bone, which surrounds the pituitary fossa and lodges its main structure, the pituitary gland (hypophysis), in area located beneath the brain in the center of the skull base. The pituitary is the gland responsible for connecting two major homeostatic systems of the body: the nervous and endocrine systems.

For a long time, Anatomists based their knowledge on the direct observation through gross dissection of embalmed cadavers. In recent decades, the incorporation of microscopic surgical techniques to perfom microdissection enhanced the knowledge of neuroanatomy and so achieved great progress in neurosurgery.

In the field of neuroimaging, MRI is the chosen diagnostic method for the study of hypothalamic-pituitary region and may provide more details of the structures that make up the central nervous system (CNS) and skull base.

This current study aims to evaluate some anatomic variations of sellar and parasellar regions and their possible differences between genders and age groups using RMIs of patients without neurological diseases. The pedriatic age group was excluded from the study.

## Methods

The study was performed at the Magnetic Resonance Unit, University Hospital, Botucatu School of Medicine, UNESP and was approved by the Medical Ethics Committee of the University of São Paulo State – UNESP.

The magnetic resonance imaging was done on a 0.5 Tesla Signa Contour scanner (GE Healthcare, Milwaukee, WI, USA), which allows thinner slices.

The images were gathered randomly from patients who underwent MRI in the period between January 2009 and July 2011.

MRI scans of the brain of males and females patients were analyzed. The age ranged between 20 and 80 years (mean age 48 years). The study included 235 females (mean age 53 years) and 145 males (mean age 40 years).

The technique for obtaining images followed the protocol used in the Unit of Magnetic Resonance Imaging Hospital of Botucatu. In all patients, five spin-echo sequences at sella turcica were performed with 1mm wide for every 0 mm of dislocation. The first sequences were sagittal T1 and coronal T2 without contrast injection. The sagittal T1 weighted (650/109 = repetition time ms/echo time ms) and image matrix was 320 × 160. The coronal T2 weighted (350/86,7 = repetition time msec/echo time msec) and matrix was 256 × 256. In all sequences, the field of view was 20 × 20 cm. Next, a dynamic sequence was made with contrast injection with 0.2 mmol/kg. The paramagnetic agent was the gadopentato de dimeglumina (Viewgam®). Postcontrast images weight T1were also obtained in sagittal and coronal planes. The protocol was in a total time of 10–15 minutes. After the acquisition, the images were transferred in DICOM format to eFilm Workstation™ .

All images were analyzed by the same radiologist with 25 years’ experience in MRI. Images of the brain and pituitary gland with lesions or expansive diseases were all discarded. In this sample, sphenoid sinus agenesis was not found.

Image analysis was performed using eFilm Workstation™ (Merge eFilm). All measurements were performed after twice image amplifications (FOV 10 × 10 cm).

**Figure 1:**
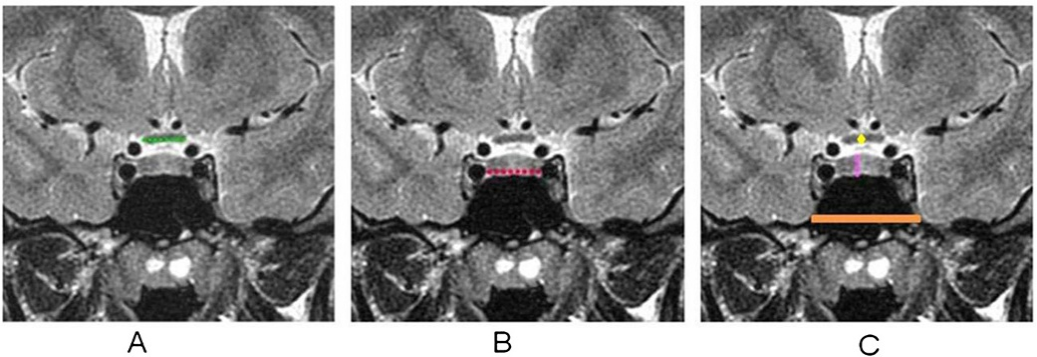
**Measurements of anatomic parameters. A**. optic chiasm width. **B**. inter-caroted distance. **C**. Hypophisis height (yellow), optic chiasm height (pink), and spenoid sinus width (orange).

The following anatomical elements were studied according to gender and age groups: optic chiasm, intra-cavernous carotid, sphenoid sinus and hypophysis. The measures selected were:

- Optic chiasm width in the most central portion in the coronal plane;
- Optic chiasm height (thickness) in the coronal plane;
- Distance between intra-cavernous segments of lateral carotid and sella turcica;
- Hypophysis: larger diameters superior-inferior (height) and latero-lateral (width) in the coronal plane;
- Sphenoid sinus width under sellar point in the coronal plane;
- Distance between the middle point of nasal columella and the anterior wall of the sphenoid sinus and the hypophysis, in the sagittal plane (columella – hypophysis and columella-sphenoid sinus distances).
  In this last item, the measured distances were performed on an imaginary axis passing through the nasal columella implantation and a point formed by the intersection of a vertical line tangent to the anterior wall of the pituitary fossa tangent to another floor in the medium sagittal plane (Figure 1).

**Figure 2:**
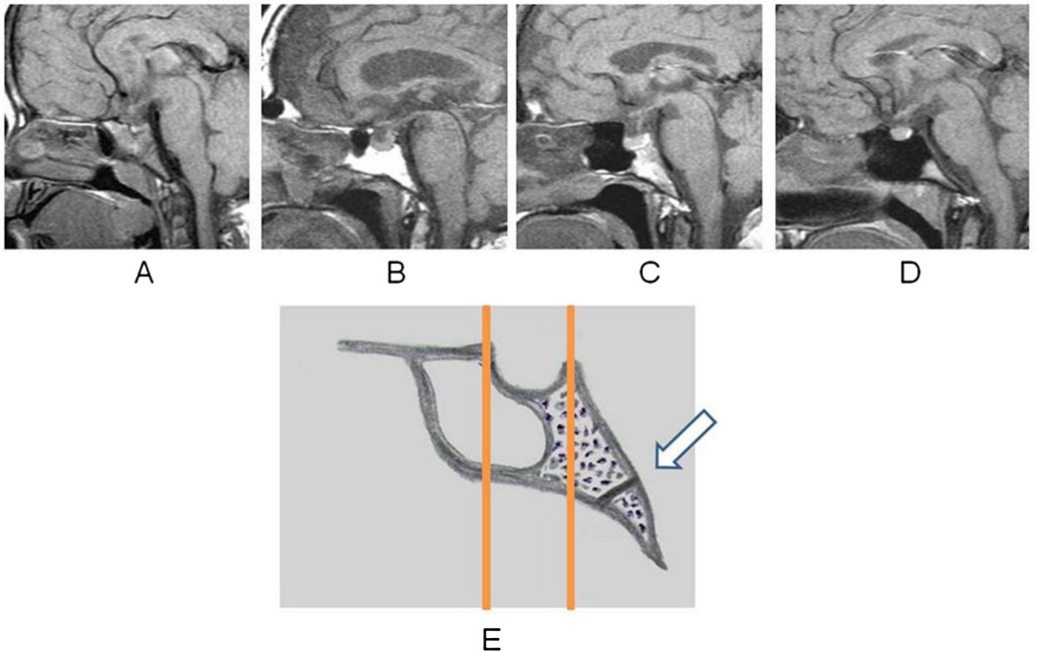
**Types of the sphenoid sinus. A**, conchal. **B**, presellar. **C**, sellar. **D**, postsellar. **E**, diagram of thwe sella and sphenoid sinus in the sagittal plane with the lines (1 and 2) used to classify types of sinus. Speno-occipital synchondrosis (arrow).

To evaluate the type of sphenoidal sinus, pneumatization and the criteria used by Guerrero^1^ were considered, excluding the semi-sellar and the conchal. Considering the sella, two imaginary lines were drawn perpendicular to the sphenoid. The 1^st^ line is tangent to the anterior pituitary fossa, and, the 2^nd^ line is tangent to the posterior boundary (Figure 2). The sinuses were classified into four types:

- Conchal – small sinus with no relation to the sella;
- Presellar - the posterior wall of the sinus reached the sella, but did not cross the 1^st^ line.
- Sellar - the posterior wall of the sinus crossed the 1^st^ line reached the floor of the sella, but did not exceed the 2^nd^ line.
- Postsellar - when pneumatization crossed the 2^nd^ line.

These dates were obtained through descriptive statistical analysis of the sample according to socio-demographic characteristics of the sphenoid sinus. Then, exploratory analyses were made of the shape of probability distributions of anatomical variables through the Shapiro-Wilk test, and comparisons between genders and age groups using the Mann–Whitney and Kruskal-Wallis tests, respectively. The conclusions about the differences and interactions were based on the values of “p” and moderated by the evidence on the subject. These results were shown in tables.

## Results

In this current study, we analyzed the MRI scans of 380 patients of 20 years and older; 38.2% are males (n = 145) and 61.8% females (n = 235). The samples were divided according to gender into 3 age groups: 20–40 years, 41–60 years and above 60 years (Table 1).

**Table 1:** **Socio-demgraphic profile of the sample (n=380)**

	n	%
Gender		
Female	235	61,8
Male	145	38,2
Age group (in year)		
20 to 40	130	37,6
41 to 60	140	34,6
61 to 80	107	28,2
Gender and age group		
Female from 20 to 40	100	26,3
Female from 41 to 60	79	20,8
Female from 61 to 80	56	14,7
Male from 20 to 40	43	11,3
Male from 41 to 60	51	13,4
Male from 61 to 80	51	13,4

In all sample, the maximum and minimum height of the pituitary gland were 3mm and 11mm respectively. No pathologies were found that could interfere with these gland dimensions. There was no significant statistical difference between the average height of the pituitary in males and females, using “Mann–Whitney and Kruskal-Wallis” tests for two independent samples. It was still noted that twenty eight women presented a gland height greater than 7mm, while only twelve men presented this value. No difference in the pituitary width was noted between both genders. Related to the women’s ages, the value of pituitary height detected U-shaped behavior (Table 2).

**Table 2:** **Medium and extreme values of the pituitary width (PW) and Sphenoid sinus width (SW), by age group (in year) and gender**

	PW			SW		
Age group	Female	Male	***p*** ^***(1)***^	Female	Male	***p*** ^***(1)***^
20 to 40	10,0	10,0	0,3	24,5	27,0	0,158
	(6,0/13,0)	(7,0/13,0) 28		(8,0/58,0)	(10,0/45,0)	
41 to 60	10,0	10,0	0,1	22,0	28,0	0,001
	(6,0/15,0)	(2,0/13,0) 03		(7,0/48,0)	(36,0/55,0)	
61 to 80	10,0	10,0	0,9	25,0	26,0	0,251
	(7,0/12,0)	(6,0/16,0) 92		(3,0/8,0)	(3,0/9,0)	
*p* ^*(2)*^	0,430	0,270	*p* ^*(2)*^	0,018	0,894	

^(1)^Mann–Whitney (independent samples).

^(2)^Kruskal-WAllis (independent samples).

The width and height of the chiasm were similar in the entire sample between the gender and age groups for the “p” of Mann–Whitney and Krusk al-Wallis tests (Table 3). For two independent samples, only one difference was detected, the disparity among women of different ages, however, it is only a discrepancy in the midst of all the other similarities. Caution is necessary to ensure that there are different age groups.

**Table 3:** **Median and extreme values of the width of the chiasm (WCHI) and the height of the optic chiasm (HCHI), by age group (in year) and gender**

	WCHI			HCHI		
Age group	Female	Male	***p*** ^***(1)***^	Female	Male	***p*** ^***(1)***^
20 to 40	12,0	12,0	0,561	3,0	3,0	0,136
	(8,0/21,0)	(8,0/17,0)		(1,0/4,0)	(2,0/4,0)	
41 to 60	12,0	12,0	0,118	3,0	3,0	0,626
	(8,0/17,0)	(8,0/16,0)		(2,0/4,0)	(2,0/4,0)	
61 to 80	12,0	12,0	<0,001	2,0	2,0	0,333
	(8,0/19,0)	(10,0/15,0)		(2,0/4,0)	(2,0/4,0)	
*p* ^*(2)*^	0,467	0,651	*p* ^*(2)*^	0,004	0,898	

The distance between the intra-cavernous segments of the internal carotid between men and women, regardless of age were similar. Indications of differences between the age groups of the same gender can be observed only among men. In this gender group, the values of intercarotid distance increase likely the age, on values of “p” using Mann–Whitney and Kruskal-Wallis tests for independent samples (Table 4).

**Table 4:** **Medium and extreme values of the inter-carotid distance (INTCAR) and pituitary height (PH), by age group (in year) and gender**

	INTCAR			PH		
Age group	Female	Male	***p*** ^***(1)***^	Female	Male	***p*** ^***(1)***^
20 to 40	16,0	16,0	0,944	6,0	6,0	0,165
	(8,0/26,0)	(8,0/27,0)		(3,0/9,0)	(3,0/9,0)	
41 to 60	15,0	17,0	0,041	6,0	5,0	0,274
	(6,0/25,0)	(8,0/29,0)		(3,0/9,0)	(3,0/11,0)	
61 to 80	17,5	19,0	0,165	6,0	5,0	0.094
	(8,0/25,0)	(10,0/31,0)		(3,0/8,0)	(3,0/9,0)	
*p* ^*(2)*^	0,037	0,058	*p* ^*(2)*^	0,030	0,055	

^(1)^Mann–Whitney (independent samples).

^(2)^Kruskal-WAllis (independent samples).

For males the mean of distances from the columella-pituitary and columella-sphenoid sinus were higher, regardless of age, but older groups did not differ either among men or women, on the values of “p” by Mann–Whitney and Kruskal -Wallis tests for independent samples (Table 5).

**Table 5:** **Median and extreme values of the distance from Sphenoid sinus to nasal columella (SCOL) and the distance from pituitary gland to nasal colomella (PCOL), by age group (in year) and gender**

	SCOL			PCOL		
Age group	Female	Male	***p*** ^***(1)***^	Female	Male	***p*** ^***(1)***^
20 to 40	66,0	77,0	<0,001	83,5	89,0	<0,001
	(55,0/80,0)	(54,0/81,0)		(72,0/95,0)	(70,0/99,0)	
41 to 60	67,0	70,0	<0,001	83,0	89,0	<0,001
	(58,0/78,0)	(61,0/78,0)		(71,0/94,0)	(76,0/99,0)	
61 to 80	66,0	73,0	<0,001	83,0	90,0	<0,001
	(58,0/77,0)	(60,0/87,0)		(70,0/95,0)	(78,0/98,0)	
*p* ^*(1)*^	0,627	0,087	*p* ^*(1)*^	0,536	0,853	

^(1)^Mann–Whitney (independent samples).

^(2)^Kruskal-WAllis (independent samples).

In the group from 41 to 60 years of age, males had larger widths of the sphenoid sinus than women, according to “p” by Mann–Whitney and Kruskal-Wallis test for independent samples. In our study considering all exams, regardless of age and gender, type post-sellas represented 58 patients (15.3%), while 300 (78.9%) had type sellar, and 22 (5.8%) pre sellar.

## Discussion

The anatomy of structures of the sellar and parasellar regions varies widely. For example, the sella turcica is described in different ways by many authors with differing values of normality. Axelsson and col (Axelsson et al. 2004) believe that these differences are due to choices of reference points and different radiological techniques.

The sphenoid sinus varies in size, shape and degree of pneumatização (Guerrero 1999; Rhoton et al. 1979; Scuderi et al. 1993; Yonetsu et al. 2000). Hamberger and col (Hamberger et al. 1961) classified within of three types: conchal, presellar and sellar depending on the pneumatization extent. Using the same criteria of classification, Rhoton (Rhoton 2002) in a study of cadavers, found the presellar type in 24% and sellar in76%. In the current study, we used a classification of Hamberger (Hamberger et al. 1961) modified by Guerrero (Guerrero 1999) but excluding semisellar and Non-pneumatized sphenoid sinus. The postsellar type represented 15.3%, while 78.9% had type sellar, 5.8% presellar.

In the sample, the width of the sphenoid sinus under the sella, showed a variation of 6–48 mm and observed that from the 41–60 year old male had widths greater than women. That is, the width of the sphenoid sinus increased with increasing age. These findings are not compatible with Yonetsu and col (Yonetsu et al. 2000) study that showed the opposite.

**Figure 3:**
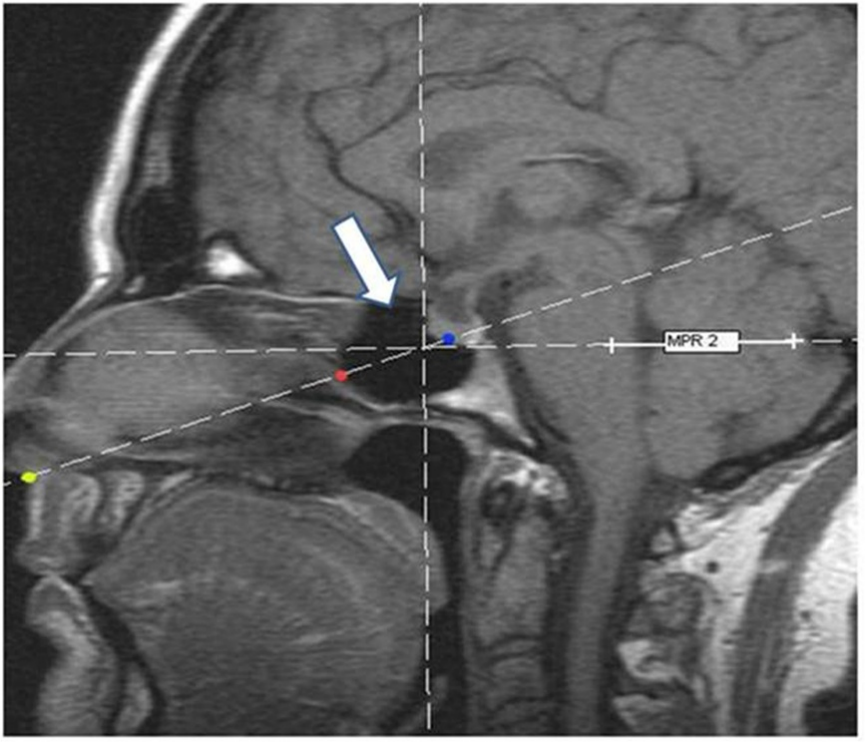
**Representation of the pre-sellar point (arrow) and the distance between columella-sphenoid and colomella -pituitary.** Implantation of the culomella (yellow). Anterior wall of the sphenoid sinus (red), hypophisis (blue).

Knowing the variations of the sphenoid sinus is crucial when trying to reach the sellar region or surrounded structures through sphenoid bone (Rhoton 2002). In this study, due to inability to locate the ostium of the sphenoid sinus on MRI, the distances measures of columella -sphenoid sinus and columella-pituitary were performed on an imaginary line that passes between the implantation point of the columella and the presellar point (Figure 3). In this way, was considered that this would be a standard of measurement. In this study, regardless of age, males presented greater distances from the nasal columella to anterior wall of sphenoid sinus and pituitary-columella than female; but age does not differ between the genders. These distances help to define the size of the specula and instruments to be used in surgery of sellar region.

The surgical approach to sellar region by trans-sphenoidal, is already an established procedure in Neurosurgery and its complications are well described by some authors (Rhoton 2002; Elias & Laws 2000; Hamid et al. 2008; Romero et al. 2012; Laws & Kern 1976). Anatomical variations of this region are the causes of complications in this procedure. The most vulnerable structures along the sinus are: the internal carotid artery near the anterior wall of the sella, and optic nerves, located superolaterally(Rhoton 2002). The study had no aim to determine whether any of these structures were exposed within the sinus.

In a study performed on cadavers Pianetti and col (Pianetti & Henriques 2000) reported that the distance from the optic chiasm to the tuberculum sella ranged from 1.5 to 8 mm (mean 4.02 ± 1.72 mm). Bergland and col (Bergland et al. 1968) described three positions to the optic chiasm: prefixed, normal-fixed and postfixed. Renn & Rhoton (Renn & Rhoton 1975) found the chiasm normal-fixed in 70% of cases, prefixed at 15% and postfixed in the remain.

The inter carotid distances in coronal plane, beside the sella, ranged from 6 to 31 mm. In cases with smaller distance, the pituitary gland showed a different conformation, similar to the findings of Rhoton (Rhoton 2002).

According to Handfas and col (Handfas et al. 2002) the pituitary gland also presents great variation in shape among different people, changing in size throughout life. Elster and col (Elster et al. 1991) reported major changes in the pituitary gland in females during puberty, pregnancy and the postpartum period, secondary to physiologic hypertrophy. In the present study, there was no significant difference in pituitary mean height between males and females.

The position of the chiasm is important in trans-sphenoidal and trans-frontal surgeries. Usually, chiasm stays prefixed or very close to the sellar tubercle blocking the passage, in both directions, between the sellar and suprasellar compartments. Alternatively, some surgeons remove the tubercle and even the sphenoidal plane (Rhoton 2002; Ciric et al. 2002; Cavallo et al. 2005). The position of the chiasm may leads to differences in the symptoms of patients with diseases in sellar and parasellar regions (Cavallo et al. 2005; Anderson et al. 1999).

Wagner and colaboradores (Wagner et al. 1997) studying magnetic resonance imaging in 123 patients, found that the width of the chiasm ranged from 8.0 to 21.0 mm (mean 14.0 ± 1.68 mm) with no differences between the genders. In this study, the width of the chiasm ranged from 8.0 to 19 mm, the height ranged from 1.0 to 4.0 mm, and was similar between genders and age group. Only one difference was detected, the difference between females of different age groups.

The height of the optic chiasm, in the present study, was homogeneous throughout the sample may reflects the limitation of this applicative to very small measures, since the eFilm Workstation software works with only one decimal place.

Among neuroimaging techniques, the MRI is the method able to provide the details of the structures of the sellar and perisselar regions. It has unique characteristics such as high sensitivity to detect subtle changes in concentration of tissue water, and high discrimination between them and multiplanar capability.

## Conclusion

Males had greater distances between the nasal columella-sphenoid sinus and nasal columella-pituitary than women, regardless of age.

The width and height of the optic chiasm were similar in gender and age groups. The Intercarotid distance and the pituitary height were similar between male and female. The width of the pituitary was similar between male and female and between age groups. In the age group of 41 to 60 years, males had larger widths of the sphenoid sinus than females. Among females, the values of the pituitary height related to age showed a U-shaped pattern. The most frequent type of pneumatization of the sphenoid sinus was the sellar.

Evaluate anatomic variations of sellar and parasellar regions and their possible differences between genders and age groups using RMIs have great importance in several medical fields. A better understanding of these complex structures is essential in clinical diagnosis and treatment of disease.
